# Comparative Content Analysis and Expert Physician Risk Assessment of Aesthetic Procedures Promoted on TikTok in Taiwan: Quantitative Study

**DOI:** 10.2196/88227

**Published:** 2026-08-19

**Authors:** Cheng-Jung Wu, Sheng-Yu Wu, Cheng-Yu Tsai, Arnab Majumdar, Jeffrey Yang, Jinn-Moon Yang, Lok-Yee Joyce Li

**Affiliations:** 1 Department of Otolaryngology, School of Medicine, College of Medicine, Taipei Medical University Taipei Taiwan; 2 Department of Otolaryngology, Shuang Ho Hospital, Taipei Medical University New Taipei Taiwan; 3 College of Biomedical Science and Engineering, National Yang Ming Chiao Tung University Hsinchu Taiwan; 4 Allergy and Rhinology, Quality HealthCare Center Taoyuan Taiwan; 5 Department of Industrial Engineering and Industrial Management, National Tsing Hua University HsinChu Taiwan; 6 Department of Civil and Environmental Engineering London United Kingdom; 7 School of Respiratory Therapy, College of Medicine, Taipei Medical University Taipei Taiwan; 8 TMU Research Center of Artificial Intelligence in Medicine and Health Taipei Taiwan; 9 Department of Biology, University of California Riverside, CA United States; 10 Institute of Bioinformatics and Systems Biology, National Yang Ming Chiao Tung University HisnChu Taiwan; 11 Department of Biological Science and Technology, National Chiao Tung University HsinChu Taiwan; 12 Department of Environmental and Occupational Medicine National Taiwan University Hospital Hsin-Chu Branch Hsinchu Taiwan; 13 School of Medicine, College of Medicine, Taipei Medical University Taipei Taiwan

**Keywords:** social media, TikTok, medical tourism, health disparities, public health, esthetic surgery, migrant health, misinformation

## Abstract

**Background:**

Algorithm-driven social media platforms such as TikTok are increasingly creating stratified layers in the cosmetic medical market, thereby influencing patient decisions and safety. In Taiwan, TikTok has 2 parallel markets: one is a formal tier promoting “cosmetic surgery tourism” to the public and the other is an underground tier targeting Southeast Asian migrant workers, providing informal, high-risk services.

**Objective:**

In this study, we quantified the clinical risks embedded within these hierarchical markets and demonstrated how digital platforms exacerbate health inequalities through algorithm-driven social media content delivery.

**Methods:**

We conducted a dual-track content analysis of 60 TikTok videos (n=30 per group). A panel of 6 specialist physicians independently evaluated the videos using the newly developed Clinical Legitimacy and Risk Scoring (CLRS) scale. This scale assesses videos across 4 dimensions: depicted environment, operator identity, risk communication, and communication channels. Interrater reliability was evaluated using Fleiss κ.

**Results:**

The CLRS was used by 6 specialist physicians and showed a significant safety difference between the two groups. The medical tourism group (model A) had an average video score of 3.2 (SD 0.7), with the primary content type of short-form videos being “surgery experience vlogs” (9/30, 30%). Concomitantly, although professionalism was apparent in these videos, underlying risks were regularly obscured. In contrast, the migrant worker group (model B) had an extremely low average score of 1.1 (SD 0.3), indicating a complete deviation from medical standards (*P*<.001) and thus posing a higher risk to patient safety. The videos in model B were mainly “home surgery demonstrations” (15/30, 50%). The interrater reliability among physicians was high (Fleiss κ=0.85; *P*<.001). The primary coding team compiled the baseline descriptive data, whereas the board-certified physician panel independently evaluated the specific CLRS metrics.

**Conclusions:**

The algorithmic architecture of TikTok creates a stratified marketplace, reinforcing socioeconomic stratification and health inequities. Specifically, regarding the informal aesthetic medical tier for migrant workers, the risk of infection has become a serious and urgent public health threat. Results of this study indicate that responsible public health interventions are urgently needed for the informal aesthetic medical tier targeting migrant workers and that there is a need to reassess TikTok’s social responsibility in reviewing high-risk medical content in short-form videos.

## Introduction

The combination of social media and medical aesthetics has created a new sales model for the advertising, awareness, and consumption patterns of medical aesthetic, surgical and nonsurgical treatments. Among social media platforms, TikTok, with its large number of users and powerful algorithm-driven short-form video distribution, has shown significant influence not only in reshaping aesthetic standards but also in driving the consumption behaviors of impressionable audiences, especially young people [1,2]. Although some studies have begun to explore the dermatologic, physiological, and psychosocial risks brought about by promoting medical cosmetic skin care trends to teenagers on these platforms, relatively little attention has been paid to how these platforms, within specific national and social contexts, facilitate the dissemination of information about and access channels for invasive medical cosmetic procedures [3,4].

This study focused on the TikTok platform in Taiwan, a society with a well-established domestic medical aesthetics industry. Although local medical aesthetic resources are widely available, 2 significant trends have emerged under the promotion of short-form videos by TikTok. The first is that medical aesthetic tourism continues to be popular among young people in Taiwan, who travel to South Korea or China for major medical aesthetic surgeries. This formal or semiformal market is characterized by groups consisting of online agents, influencers, and clinics that use TikTok to promote all-inclusive surgical packages. The second trend is more covert, existing within Taiwan’s community of more than 830,000 Southeast Asian migrant workers, which also includes an estimated 90,000 undocumented migrant workers [5,6]. Because of multiple obstacles such as language barriers, economic limitations, and limited access to formal medical aesthetic channels in Taiwan, an underground medical aesthetic market has flourished. There is some promotion of this informal market on TikTok, but promotion mainly occurs through closed networks with specific language use, providing medical aesthetic services such as injectable fillers or thread lifting performed by unlicensed practitioners (usually fellow citizens in the beauty industry) in nonclinical settings.

Traditionally, tools such as DISCERN have long been used as the gold standard for assessing the quality of consumer health information. The DISCERN instrument is a widely used health information evaluation tool, jointly developed by the University of Oxford in the United Kingdom and patient organizations. It is designed to help patients, caregivers, and the public evaluate the quality and reliability of publications or website content related to health care and treatment options. However, DISCERN was fundamentally designed for long-form texts and websites. In modern times, applying DISCERN to fast-paced, algorithm-driven short-form videos creates significant structural gaps, and it is not suitable for evaluating such media. DISCERN often fails to capture critical multimedia dynamics, such as visual environments, unverified operator identities, and real-time interactive communication channels, which are characteristic of modern social media promotional methods. Therefore, as a new, specialized tool is needed to adapt to the changing times, we developed the new Clinical Legitimacy and Risk Scoring (CLRS) scale to assess the safety of these health care–related short-form video platforms.

The TikTok platform has developed algorithms aimed at maximizing user engagement. These algorithms inevitably isolate the two distinct demographic groups mentioned above, resulting in two parallel and unique medical aesthetics ecosystems [3]. From a physician’s perspective, we assumed that both ecosystems harbor inherent risks. However, the medical aesthetics risks and health hazards associated with these two ecosystems differ in nature and severity. This outcome exacerbates social stratification and health inequalities. This is the first study to provide a scoring method based on the CLRS for these two demographic groups and conduct a comparative content analysis and expert risk assessments of videos aimed at these two groups on Taiwan’s TikTok. In this research, video types were systematically classified and risk scores were quantified and verified by a panel of 6 specialist physicians. This study aimed to analyze the degree of threat to public health posed by these two parallel cosmetic medicine markets, elucidate the mechanisms by which social media algorithms operationalize health disparities among marginalized groups, and test the hypothesis that algorithmic personalization actively directs disadvantaged groups toward dangerous, unregulated medical practices.

## Methods

### Overview

For this study, we established two completely new and entirely different TikTok accounts to simulate real user settings. We applied a systematic search strategy using different high-traffic hashtags for these 2 new accounts [3].

Account A (medical tourism track; model A) was set up to represent a young Taiwanese woman interested in plastic surgery and cosmetic medical treatments. The user interface was set to traditional Chinese. This account was used to actively search for and interact with relevant content using hashtags such as “#KoreanPlasticSurgery” and “#MainlandCosmetic.”

Account B (informal migrant worker track; model B) was set up to represent a Southeast Asian female migrant worker working in Taiwan. The user interface was set to Vietnamese. The hashtags and keywords used for searching included “#fillerTaiwan,” “#CosmeticTaiwan,” and “#suntikputihTaiwan.”

For both accounts, researchers repeatedly liked and saved relevant medical aesthetics videos to train TikTok’s “For You” page algorithm. From May 2025 to July 2025, 60 independent videos with the highest view counts were collected, including 30 (50%) videos from model A and 30 (50%) videos from model B. To ensure content diversity, only 1 video per creator was included. The inclusion and exclusion criteria were strictly enforced. Videos were included if they were 15 to 600 seconds in length, contained specific medical advice or explicit price promotions, and had been posted within the past 12 months. Videos were excluded if they were duplicates or lacked real human elements (eg, static slideshow presentations without integrated dynamic audiovisual content).

### Ethical Considerations

The study protocol was reviewed and approved by the Taipei Medical University Joint Institutional Review Board (TMU-JIRB; N202511073).

### Development and Validation of the CLRS

The development of the CLRS tool used a deductive-inductive hybrid approach. In terms of deduction, the tool drew on core bioethical principles, such as the principle of nonmaleficence, as well as local medical advertising regulations (eg, Taiwan’s Medical Care Act) to establish benchmark standards for medical legality.

In terms of induction, through a preliminary analysis of 60 nonsample medical aesthetics videos, we found that traditional tools such as DISCERN were not suitable for evaluating short-form medical videos. DISCERN’s evaluation methods overlooked certain phenomena in new social media, such as cross-border medical intermediaries, ultra–low-cost package deals, and invasive procedures conducted in home environments, which cannot be assessed using traditional DISCERN. These insights were transformed into specific indicators across 4 key dimensions to quantify platform-specific safety signals. The CLRS was specifically designed for this study to address the lack of visually oriented tools for assessing the quality of short-form videos. However, the research team anticipates that this scale will have strong practical utility and will be widely applied to various medical topics and short-form video platforms in the future.

### Data Coding and Analysis

#### Video Genre Classification

Drawing on methodologies used to analyze social media content, we classified videos into different “genres” based on common narrative formats. The analysis phase was also a multilevel evaluation. Initially, the primary coding team, composed of 2 independent researchers, watched the videos and outlined the baseline characteristics of each short-form video, while classifying each video as model A or model B. The researchers conducted a comprehensive assessment of the short-form videos, including audio and visual elements, and on-screen subtitles, rather than relying solely on the textual transcripts. Although the content targeted 2 different language groups (traditional Chinese and Vietnamese), both coders were core members of the research team. They used a single, unified coding book and received synchronized consensus training to ensure consistency. Any disagreements in classification were discussed and resolved through a consensus meeting led by a senior consulting physician. In this study, the primary coded variables included promotion model (A vs B), video type (eg, surgical procedure vlog and home treatment demonstration), and baseline indicators. These variables were mapped before conducting clinical evaluations to establish a descriptive framework for the platform.

#### Expert Physician Review and Risk Scoring

To critically quantify the clinical risks brought about by TikTok videos, a group of specialist physicians was recruited as the expert panel. The panel consisted of 2 dermatologists, 2 facial plastic surgeons, and 2 plastic surgeons, each with more than 10 years of clinical experience in aesthetic medicine. In this single-blind study, panel members were unaware of the study hypotheses and the dual-track study design. Each video was viewed at least twice, after which the panel members rated it using the innovative standardized CLRS tool.

The 6-member expert panel rated videos using digital CLRS rating cards built into a securely encrypted Google Forms interface. The CLRS assessment covers 4 domains, namely environment, operator identity, risk communication, and communication channels. Scores for each domain ranged from 1 point (highest risk and lowest legitimacy) to 5 points (lowest risk and highest legitimacy). The final rating was calculated as the average of these 4 scores (Table 1). This digital rating system eliminated transcription errors during the data conversion process, thereby assuring data accuracy.

**Table 1:** Clinical Legitimacy and Risk Scoring system.

Scores	Domain 1: depicted environment	Domain 2: operator identity	Domain 3: risk communication	Domain 4: communication channel
1 (highest risk)	Private residence, hotel room, or back room of a nonmedical business	Unspecified peer (eg, “sister” or “friend”) or self-proclaimed beautician with no credentials shown	Active misinformation (eg, “100% safe” or “no side effects”)	Encrypted, anonymous private chat (eg, Telegram or WhatsApp)
2	Unspecified or ambiguous location	Vaguely described as “experienced” or “professional,” without evidence	Complete absence of any mention of risk, pain, or recovery	Private social media direct messages (eg, TikTok or Instagram)
3	Appears to be a private beauty salon or spa rather than a medical clinic	Presented as a nurse or technician, but credentials are not verifiable	Mentions minor, transient side effects only (eg, “a little swelling”)	Public-facing but personal social media account
4	Appears to be a formal medical clinic, but accreditation is not specified	Presented as a physician, but the name or credentials are difficult to verify	Acknowledges a recovery period and common side effects	Official business page on a social media platform (eg, Facebook Business Page)
5 (lowest risk)	Appears to be an accredited hospital or major surgical center	Presented as a named, board-certified surgeon with verifiable credentials	Mentions specific serious potential complications and contraindications	Official clinic or hospital website with publicly available contact information

### Statistical Analysis

Descriptive statistics were used to summarize the video characteristics. Mean scores of the CLRS for each model were calculated based on the average ratings from the 6 board-certified physicians. Interrater reliability for the CLRS was assessed using Fleiss κ. Group differences in mean CLRS total scores and domain scores between models A and B were evaluated using the Mann-Whitney *U* test, given the ordinal nature of the scoring scale and the nonnormal distribution of scores. A 2-tailed *P* value of <.05 was considered statistically significant. All statistical analyses were performed using SPSS (version 26.0; IBM Corp).

## Results

### General Characteristics and Promotional Strategies

An analysis of videos on TikTok showed that the promotional market for medical aesthetic videos clearly has distinct tiers. Promotion system model A is built on narratives of the “aspiration to become beautiful,” using testimonials from influencers or celebrities and the legitimacy of clinical medical environments. Model B, on the other hand, is a completely different setting. The strategy of model B is rooted in leveraging trust within peer communities while avoiding legal medical environments and placing more emphasis on convenience, extreme cost-effectiveness, and privacy. Key differences are summarized in Table 2.

**Table 2:** Comparative analysis of 2 tiers of aesthetic procedure promotion on TikTok.

Comparison metrics	Model A: medical tourism (to China or South Korea)	Model B: informal procedures (for migrant workers)
Promotional strategy	Aspirational results, influencer testimonials, luxurious clinic environments, and package deals	In-group trust (“by us, for us”), extreme affordability, privacy, and the “at-home” service convenience
Common procedures	Invasive surgeries, including rhinoplasty, blepharoplasty, orthognathic (jaw) surgery, and breast augmentation	Minimally invasive procedures, including dermal filler injections (nose, lips, and chin), thread lifts, and “whitening” injections
Cost and payment	High cost, often presented as “value” packages (US $5000-$15,000); payment through formal channels	Extremely low cost, often priced per unit (eg, US $100-$300 per syringe); payment typically made in cash
Risk communication	Minimal; focuses on the recovery period, while severe complications are rarely mentioned	None; often includes explicit, false claims such as “100% safe” and “no side effects”
Depicted environment	Modern, brightly lit clinics and hospital operating rooms	Private residences, hotel rooms, or back rooms of other businesses
Operator identity	Presented as board-certified, experienced surgeons and medical teams	Presented as a fellow migrant worker, a “beautician,” or an experienced peer
Communication channel	Public-facing business accounts (eg, official LINE, WeChat, or Instagram accounts)	Encrypted, private messaging apps (eg, WhatsApp or Telegram) or private Facebook groups

### Video Genre Distribution

After categorizing the videos by type, 2 different narrative styles were detected (Table 3). Model A mainly used a “surgical journey vlog” format (9/30, 30%), which promotes a parasocial connection between influencers and viewers, making the surgical experience more personal [7,8]. In contrast, model B videos often feature “surgical demonstrations in personal studios” (15/30, 50%), thereby normalizing medical aesthetic procedures performed in unsafe environments.

**Table 3:** Distribution of video genres by promotion model (N=60).

Genres	Model A: medical tourism (n=30), n (%)	Model B: informal procedures (n=30), n (%)
Surgery journey vlog	9 (30)	0 (0)
Clinic promotion or tour	7 (23.3)	0 (0)
Agent question and answer	5 (16.7)	0 (0)
Dramatic transformation	6 (20)	4 (13.3)
At-home procedure demonstration	0 (0)	15 (50)
Client testimonial	1 (3.3)	8 (26.7)
Product or service advertisement	2 (6.7)	3 (10)

**Physician-Rated CLRS**

Six board-certified physicians used the CLRS system to evaluate the 60 videos. Scores were assigned to promote the evaluation of these two different types of videos. The CLRS provides a quantitative measure of the safety differences between the videos. The average total score for model A videos was 3.2 (SD 0.7), while the average total score for model B videos was extremely low at 1.1 (SD 0.3). The interrater reliability among the 6 expert physicians was significant (Fleiss κ=0.85; *P*<.001), indicating a high level of consistency in risk assessment.

The CLRS analysis revealed the specific risk areas for each model (Table 4). Model A short-form videos scored highest in the “depicted environment” domain (mean 4.1, SD 0.6), successfully shaping the image of clinical medical professionals. However, this group scored lowest in the “risk communication” domain (mean 2.2, SD 0.7), confirming that the video failed to adequately inform patients about serious complications that may arise from surgery or treatment. Model B videos scored the highest risk level across all 4 domains, with the lowest scores in the “operator identity” and “risk communication” domains (mean 1.0, SD 0.2 and mean 1.0, SD 0.1, respectively). This finding indicates that these videos completely and systematically deviated from any standards of medical quality and safety.

**Table 4:** Mean physician-rated Clinical Legitimacy and Risk Scoring (CLRS) by domain and promotion model.

CLRS domains	Domain 1: depicted environment	Domain 2: operator identity	Domain 3: risk communication	Domain 4: communication channel	Overall CLRS score
Model A: medical tourism, mean (SD)	4.1 (0.6)	3.8 (0.9)	2.2 (0.7)	2.8 (0.7)	3.2 (0.7)
Model B: informal procedures, mean (SD)	1.1 (0.3)	1.0 (0.2)	1.0 (0.1)	1.3 (0.5)	1.1 (0.3)

## Discussion

### Principal Findings

As confirmed by this study, the innovative CLRS system can integrate platform-specific features (eg, communication encryption and environmental tracking) into quantifiable metrics, successfully filling the gap in DISCERN’s analysis of short-form videos. In the context of modern social media, the CLRS, as a fast and visual filtering mechanism, can effectively identify potentially dangerous peer-to-peer medical advertising, thereby complementing DISCERN. DISCERN is the gold standard for assessing the quality of health information; however, because TikTok is a visually oriented platform and its videos are short form, the traditional long-form DISCERN assessment tool is not entirely applicable. Therefore, we referred to the core principles of DISCERN and developed the CLRS by synthesizing established health communication standards, such as the DISCERN instrument and the American Medical Association (AMA) benchmarks (X-9), while adapting them to the unique characteristics of short-form video platforms. The CLRS was developed using a deductive-inductive hybrid approach. The deductive method refers to the principle of nonmaleficence in medical ethics and clinical guidelines (eg, Taiwan’s Ministry of Health and Welfare regulations on medical aesthetics advertising), which define what constitutes high-risk behaviors. The other method was inductive, which was used during a pilot study of 60 videos, in which phenomena not captured by existing scales were discovered, such as inducing cross-border medical treatment, presenting low-cost promotional packages, and emphasizing surgeries in nonmedical settings.

From these observations, specific subitems of the CLRS were inductively derived. The “clinical legitimacy” domain draws on regulatory standards for medical advertising (eg, local regulatory reference), while the “risk” domain was inductively derived from thematic analyses of prevalent aesthetic procedure promotion on TikTok, focusing on safety signals that DISCERN may overlook, such as the glamorization of invasive procedures in nonclinical settings.

The main findings of this study indicate that TikTok in Taiwan is not a single source of medical information but rather a stratified platform in which access to cosmetic medical procedures and related risks is heavily influenced by socioeconomic status, ethnicity, and legal identity. Paradoxically, although such platforms are expected to enhance information transparency and equalize the cost of cosmetic medical procedures, our analysis showed the opposite. Instead, the platform fosters a more polarized and opaque market, intensifying medical risks caused by information asymmetry. These 2 tiers present fundamentally different types of risks, a finding supported by empirical evidence from the quantitative risk assessment and highly validated by a panel of expert physicians.

In our study, these 2 quantified risks, information asymmetry and physical threats, represent fundamentally different categories of risk across 2 tiers. The CLRS quantitative risk assessment table was validated by a strong consensus from a panel of medical experts, providing an empirical medical basis for this conclusion. The risk associated with model A mainly lies in the quality of care and information asymmetry. Therefore, patients participating in medical aesthetic tourism face significant risks in verifying physicians’ qualifications and managing postoperative care [9]. The risk associated with model B pertains to immediate patient safety. Its CLRS score was as low as 1.1, confirming that it represents a severe public health crisis and risk of infection. The risks are urgent and multiple: practitioners without a medical background could cause catastrophic complications such as vascular embolism and severe infections [10]; there is a high chance of using illegal and nonsterile counterfeit products, leading to serious local infections and inflammation [10]; procedures performed in unhygienic environments pose a very high risk of infection and transmission of bloodborne pathogens; and the staff in this unit completely lack the ability to handle emergency complications.

The divide between these two tiers on TikTok is a direct manifestation of the broader social class disparities and health inequalities within the health care ecosystem. TikTok’s digital stratification has already shifted from a virtual interface to a tangible expression of social class differences, health inequities, and socioeconomic gaps. In this study, the divide between these two tiers on TikTok in cosmetic medicine is a direct reflection of structural inequalities. The trend toward medical aesthetic tourism (model A) is jointly driven by medical intermediary agencies and globally minded consumers influenced by transnational media flows [1]. The emergence of the model B market is a direct expression of these structural inequalities. It is a form of “health care” that arises not because it is explicitly prohibited by the formal system but because structural barriers (eg, high costs, language barriers, and the well-founded fear of deportation among undocumented migrant workers when interacting with any formal institution) make straightforward medical aesthetics unattainable. Due to high costs, language barriers, or fear of immigration authorities, migrant workers may be unable to access aesthetic treatments through Taiwan’s formal medical system [6,11]. The rise of this underground market indicates how marginalized groups, when structurally excluded from the formal medical aesthetic system, use peer networks to meet these aesthetic needs, even if it comes at extremely dangerous health risks. The emergence of this underground economy fills a gap and demonstrates how marginalized groups use flexible methods to fulfill these aesthetic needs when the formal system fails to reach them, despite exceedingly dangerous consequences. Short-form videos on TikTok have become a normalized channel for disseminating these risks.

Local health authorities in Taiwan face a dual challenge and contradictions of punitive regulatory enforcement, in terms of the government facing challenges in monitoring advertisements for cross-border medical services (model A) and domestic unlicensed medical practices (model B). These challenges highlight the regulatory failure of health authorities. Although authorities have established support systems for workers, such as the 1955 hotline, the main enforcement mechanism against undocumented migrant workers remains largely punitive. However, from a public health perspective, these punitive measures may have counterproductive effects. By increasing the risk of punishment when discovered, these measures may exacerbate undocumented workers’ fear of any interaction with official health care units in Taiwan. This fear, combined with targeted short-form video content on TikTok, may push them further toward underground unlicensed medical aesthetic providers. Immigration enforcement may unintentionally worsen this public health crisis. This also raises key questions about platform responsibility. The way algorithms are designed may create short-form video spaces that normalize high-risk behaviors. Platforms are not neutral providers of medical aesthetic content but active participants in creating and sustaining these high-risk medical aesthetic markets.

This study has several limitations. First, a limited number of videos were selected through TikTok’s algorithm, which may not represent the full picture of content on the TikTok platform. In addition, because models A and B represent simulations using only a single account, the results may reflect their individual algorithmic paths, rather than patterns that can be consistently reproduced across different users. Given that TikTok’s recommendation engine is highly personalized and opaque, relying on a single simulated account to test models A and B produces localized trajectories. This lack of sample diversity makes it impossible to generalize the findings to a broader user base or reliably replicate the dataset in future studies. Moreover, the specific content pushed by the social media platform will change over time and cannot be directly reproduced.

Future research should analyze multiple accounts representing different demographic groups to better account for algorithmic variability. Second, this analysis was based solely on video content, and it could not measure the actual incidence of real-world complications. Third, this was a cross-sectional study, which could not evaluate the longitudinal results produced by the continuous contact algorithm. To address this limitation, future studies should adopt a hybrid research design incorporating longitudinal follow-up. Furthermore, in-depth qualitative interviews with users represented by model A and model B may provide deeper insight into their motivations and subsequent clinical complications.

### Conclusions

Social media platforms such as TikTok have gone beyond mere information dissemination; their algorithms enhance individual engagement with the platform through carefully curated personalization. These algorithms may, in turn, strengthen and exacerbate existing socioeconomic inequalities. In this study, the CLRS analysis verified by specialist physicians indicated that this digital stratification actively amplifies risks associated with medical aesthetics. For the public, the platform serves as a gateway to international medical tourism while concealing critical postoperative care and infection risks. For marginalized migrant worker communities, it acts as an efficient channel, guiding them toward unregulated, potentially life-threatening medical aesthetic procedures.

Ultimately, this study issues an urgent, data-driven call to action for Taiwan’s public health authorities. Rather than relying solely on traditional, punitive prevention, a more proactive harm reduction strategy should be implemented. To be specific, effective policies must combine culturally tailored, multilingual health outreach with targeted administrative reforms. These approaches may contribute to dismantling the systemic barriers that prevent migrant workers from accessing professional medical aesthetic services in Taiwan. Furthermore, a paradigm shift regarding “platform accountability” is imperative. When algorithms explicitly craft a lucrative market for fatal medical negligence targeting vulnerable groups, the platform ceases to be a neutral medium. Future regulatory frameworks must compel social media corporations to take responsibility for the foreseeable public health harms propagated by their algorithmic architectures.

## Data Availability

The data presented in this study are available from the corresponding author upon reasonable request.

## Abbreviations

AMA: American Medical Association
CLRS: Clinical Legitimacy and Risk Scoring
TMU-JIRB: Taipei Medical University Joint Institutional Review Board
